# The Influence of Image Exposure Duration on Facial Perception—Why It Matters

**DOI:** 10.1111/jocd.70457

**Published:** 2025-09-16

**Authors:** Michael Alfertshofer, Sebastian Cotofana, Jeremy B. Green, Leonard Knoedler, Rod J. Rohrich, Kristina Davidovic, Sachin M. Shridharani, Carlos Bravo, Samuel Knoedler, Boguslaw Antoszewski, Joanna Kempa, Anna Kasielska‐Trojan

**Affiliations:** ^1^ Department of Oromaxillofacial Surgery University Hospital LMU Munich Germany; ^2^ Department of Plastic and Hand Surgery, Klinikum Rechts der Isar Technical University Munich Munich Germany; ^3^ Department of Dermatology Erasmus Medical Centre Rotterdam the Netherlands; ^4^ Centre for Cutaneous Research, Blizard Institute Queen Mary University of London London UK; ^5^ Department of Plastic and Reconstructive Surgery Guangdong Second Provincial General Hospital Guangzhou Guangdong Province China; ^6^ Skin Associates of South Florida and Skin Research Institute Coral Gables Florida USA; ^7^ Division of Plastic and Reconstructive Surgery Harvard Medical School, Massachusetts General Hospital Boston Massachusetts USA; ^8^ Private Practice Dallas Texas USA; ^9^ Center for Radiology and Magnetic Resonance Imaging Clinical Center of Serbia Belgrade Serbia; ^10^ Division of Plastic and Reconstructive Surgery Washington University St. Luis Missouri USA; ^11^ Private Practice New York New York USA; ^12^ Private Practice San José Costa Rica; ^13^ Plastic, Reconstructive and Aesthetic Surgery Clinic, Institute of Surgery Medical University of Lodz Lodz Poland; ^14^ Individual Course of Study in Plastic, Reconstructive and Aesthetic Surgery Clinic, Institute of Surgery Medical University of Lodz Lodz Poland

**Keywords:** eye tracking analyses, facial aging, facial beauty, facial perception, facial regions

## Abstract

**Background:**

First impressions from faces, thought to serve an adaptive evolutionary role, vary by age and gender. Understanding gaze patterns is key to uncovering how these impressions form. Understanding facial gaze during brief (1 s) and longer (8 s) exposures helps identify key facial areas and behaviors, providing insights for better aesthetic assessment for non‐invasive and surgical procedures to enhance outcomes and satisfaction.

**Aims:**

To evaluate whether we perceive faces differently depending on the gaze duration.

**Methods:**

The number of stable eye fixations (total fixation count) of *n* = 55 observers (34 females, 21 males) in seven facial regions of four visual stimulus images (young and old male and female) was analyzed using eye‐tracking technology. The four visual stimulus images were presented to the observers twice in a randomized order with exposure times of 1 and 8 s.

**Results:**

Regardless of age or sex of the face, fixation rankings followed similar patterns, with slight shifts between 1 and 8‐s exposures. Age‐ and sex‐specific analyses revealed gaze pattern differences.

**Conclusion:**

A consistent facial scanning pattern, starting with central features (periorbital, nose, perioral) and expanding to cheeks, forehead, chin, and jawline, was observed regardless of the inspection time. However, longer image exposure shifts focus to regions more crucial for facial expressions, such as the perioral and forehead areas. This effect is especially visible for mature faces in which increased exposure time makes observer focus on the areas where age‐related changes are most visible (i.e., perioral) and, on the other hand, possible to be corrected with injections.

## 1 Introduction

The facial aesthetic industry is continuously growing due to the high demand for aesthetic surgical and non‐surgical treatments in today's society [1, 2]. The aesthetic needs of patients predominantly focus on the amelioration of facial aging signs, which include the smoothing of rhytids and wrinkles, repositioning of sagging facial soft tissues, and/or replenishing lost facial volume in various facial regions like cheeks and lips [3, 4, 5]. However, in younger individuals entering the facial aesthetic field earlier, there is a growing preference for minimally invasive and more subtle treatments. This new set of patients favors short downtimes, less expensive procedures, and the absence of a stigma of having received an aesthetic treatment, thereby shifting the aesthetic portfolio of providers from surgical to minimally invasive.

The variety of treatment requests consequently demands accurate patient assessment, effective communication, and guidance towards the most suitable treatment strategy. Due to the lack of objective, universally available assessment tools and outcome measurements, the aesthetic treatment providers are often left to independently evaluate the aesthetic status quo and determine the starting and ending points of a patient's individual aesthetic journey.

This independent evaluation is prone to a wide array of evaluation and cognition biases including—but not limited to—the perception drift [6, 7], halo effect [8, 9, 10], or the central tendency bias [11] to name just a few of many biases that have been shown to influence human decision making. The aesthetic field is not exempt from such biases which potentially can influence patient assessment, treatment selection, and ultimately the aesthetic outcome. When it comes to assessing beauty prior to aesthetic treatments, previous eye tracking‐based investigations have identified that humans have an internal blueprint for beauty which enables them to determine within milliseconds whether an inspected object (or face) is perceived as beautiful (or not) [12, 13, 14, 15, 16]. This process can be compared to a children's shape sorting game: the inspected object (or face) either matches into the respective shape (or internal beauty blueprint) or not. If the object matches, it is classified as beautiful, and the observer can move on to the inspection of the next object (or the next facial feature). If the object does not match, the observer tries to make it fit (see shape sorting game) which requires additional visual information about the inspected object. In eye tracking language, this non‐matching scenario is represented by longer viewing times.

Face perception is, however, specific as it has an important social and evolutionary role. Impressions from faces are fast and automatic, elicited by even brief exposure—100 ms or less [17]. Such a fast gaze is focused on sex and age‐related features, as these features play the most important role. However, little is known about the change in perception between a first impression and when a face can be inspected for a longer duration. It could be assumed that during the initial inspection of a face, which may last approximately up to 1 s, specific facial features or aesthetic needs are visually attended to. However, it is also possible that the same facial features and aesthetic needs identified in the initial brief moment are those that would be attended to during longer inspection times. The answer to this interesting question is relevant for patient assessment in every aesthetic provider's consultation, as well as to a patient's perception of their own face before or after an aesthetic treatment.

Therefore, the objective of this eye tracking study is to investigate the gaze pattern of observers when faces of varying aesthetic features (young vs. mature; male vs. female) are presented for a short (=1 s) versus a longer (=8 s) image exposure time. The results of the gaze pattern will allow identifying clinically relevant facial regions and gaze behavior which can be used to guide aesthetic assessment strategies.

## 2 Material and Methods

### 2.1 Study Sample

In this eye‐tracking study, a total of *n* = 55 participants with medical and non‐medical backgrounds were included following their recruitment among out‐patients, medical staff, and non‐medical staff. All study participants were of Polish origin and had a Caucasian ethnic background. The study was carried out between January and February 2024 at the Plastic, Reconstructive and Aesthetic Surgery Clinic of the Medical University of Łódź, Poland. Ethical approval was obtained from the Bioethics Committee of the Medical University of Łódź prior to study initiation, with the protocol number RNN/217/22/KE.

### 2.2 Visual Stimulus Images

**FIGURE 1:**
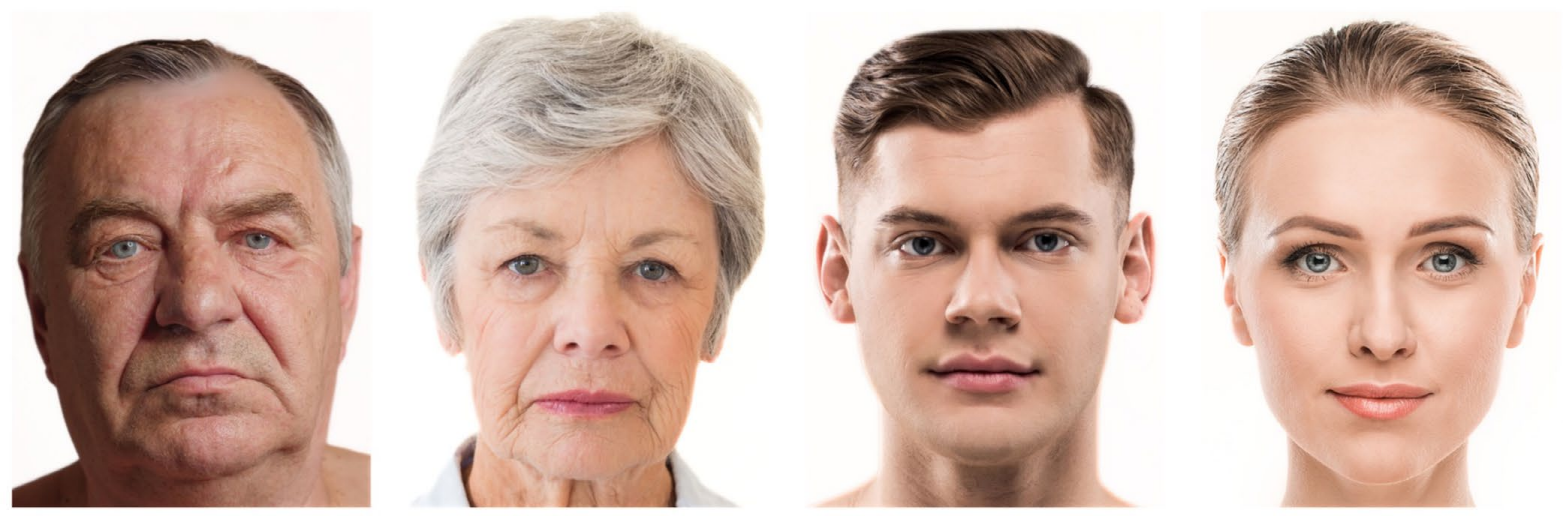
The four visual stimulus images in frontal view observed in this study: Mature male, mature female, young male, and young female.

The *n* = 55 study participants (=observers) were asked to look at four facial images of Caucasian faces shown on a 21.5‐in. screen. These four images (termed the visual stimulus images) showcased the following faces in a frontal view: mature male, mature female, young male, and young female. (Figure 1) The four visual stimulus images were presented to the observers in random order twice: 1 s image exposure interval and 8 s image exposure interval. The sequence of the visual stimulus images (sex, age) and image exposure time (1 vs. 8 s) was randomized, resulting in blinding of the observers to visual stimulus type and exposure time. This resulted in the specific study design in which the observers had no preparation regarding whether they would be able to inspect any of the randomized four images for 1 s or for 8 s. Between each visual stimulus presentation, a break interval of 5 s was provided showing a black screen. Images for the visual stimulus were chosen from an Adobe Stock database (Adobe System Inc., San Jose, California).

### 2.3 Eye Movement Analysis

**FIGURE 2:**
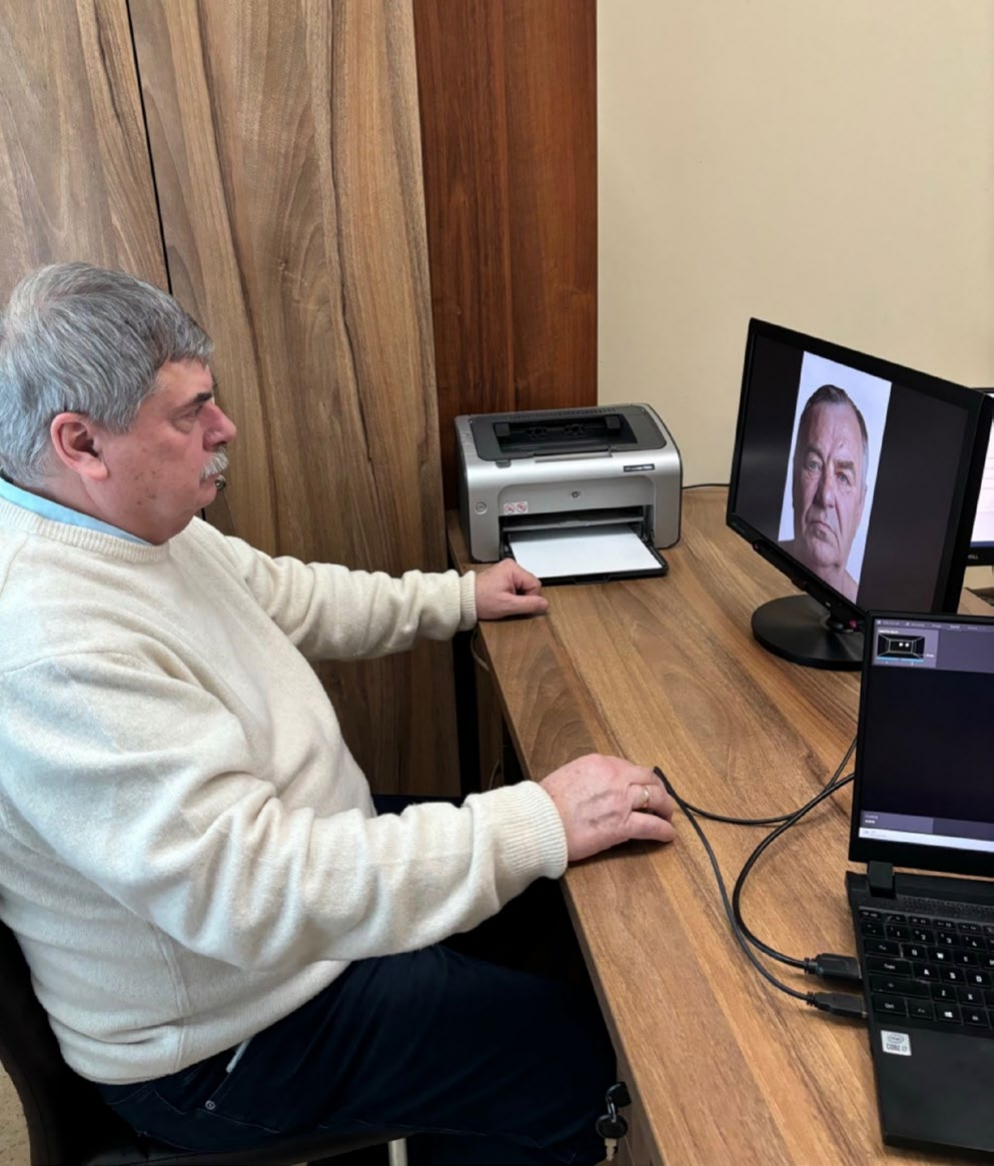
Observers were seated at a standardized and consistent distance from a 21.5 in. monitor displaying the visual stimulus images. The eye‐tracking equipment was attached to the monitor to capture the gaze pattern.

During the exposure of the four frontal facial images, the gaze pattern and the count of stable eye fixations per facial region were recorded for each observer. For this task, the eye‐tracking system Tobii Pro Spark (Tobii, Danderyd, Sweden) was employed. The system was attached to the frame of a 21.5‐in. monitor ASUS VS229HV (ASUS, Taipei, Taiwan) which displayed the visual stimulus images as described previously [12]. (Figure 2) Each observer was positioned at a standardized distance of 60–80 cm in front of the monitor to allow for equal conditions during the eye tracking analyses. The recorded data was processed and analyzed via the proprietary software Tobii Pro Lab program (Tobii, Danderyd, Sweden).

**FIGURE 3:**
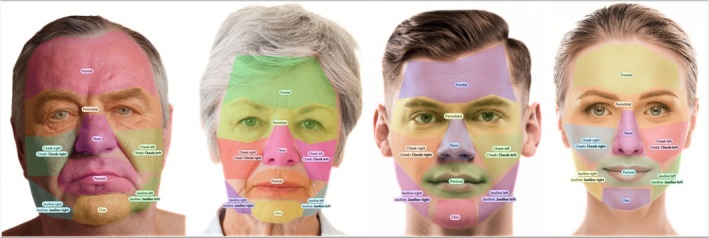
The following facial regions were defined and analyzed in our study, independent of age and sex of the visual stimulus image: Periorbital, nose, perioral, cheek, frontal, chin, and jawline.

A stable eye fixation was defined as a stable gaze towards one facial region lasting longer than 0.08 s. The number of stable eye fixations per predefined facial regions was used as the primary parameter of this investigation. The following facial regions were a priori determined for each of four facial images: periorbital, nose, perioral, cheek, frontal, chin, and jawline (Figure 3).

### 2.4 Statistical Analysis

The analytic procedure of the study is based on the assumption that more relevant facial regions will receive more attention per time and therefore will display a higher fixation count (=more stable eye fixations during the interval in which the visual stimulus image is displayed). On the contrary, less relevant facial regions will have a lower fixation count per visual stimulus presentation interval. Varying the visual stimulus exposure interval between 1 and 8 s allows one to identify more versus less relevant facial regions.

Measurements of bilateral facial regions (e.g., cheek, jawline) were averaged and their respective mean value is reported. All calculations were run using SPSS Statistics 27 (IBM, Armonk, NY, USA) and differences were considered statistically significant at a probability level of ≤ 0.05 to guide conclusions. Results are presented as mean values and their respective 1× standard deviation: (mean [SD]).

## 3 Results

### 3.1 Study Sample

A total of *n* = 55 observers (34 females, 21 males) with a mean age of 52.8 ± 17.4 years [range: 18–86] were included in this eye‐tracking analysis. The observers included individuals of medical (*n* = 12; 21.8%) (physicians, medical students, nurses, hospital workers, and allied healthcare professionals) and non‐medical backgrounds (*n* = 43; 78.2%). None of the observers had a professional background in plastic surgery or aesthetic medicine.

### 3.2 Overall Analysis

**FIGURE 4:**
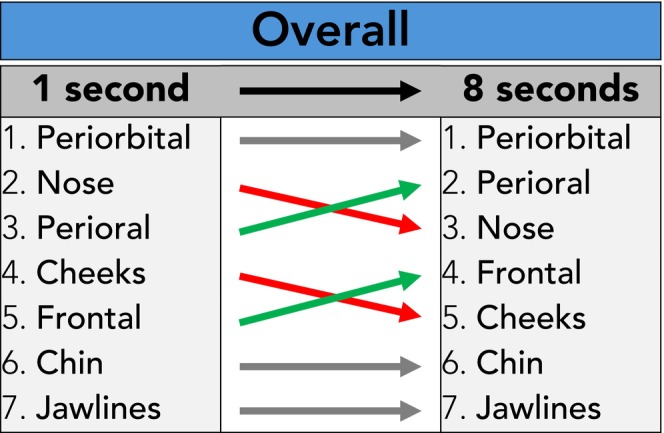
Overall comparison of total fixation counts per facial region in 1‐s versus 8‐s image exposure intervals, independent of age and sex of the visual stimulus image.

Independent of the gender or age of the visual stimulus image presented, the following ranking was identified during the 1 s image exposure interval (ranked by fixation count): periorbital 2.33 (1.0), nose 0.59 (0.6), perioral 0.35 (0.8), cheeks 0.19 (0.3), frontal 0.11 (0.2), chin 0.03 (0.1), and jawlines 0.01 (0.1). When presenting the visual stimulus for the duration of 8 s, the following ranking was recorded: periorbital 12.24 (3.8), perioral 3.10 (2.8), nose 3.01 (2.8), frontal 1.70 (2.2), cheeks 0.98 (1.1), chin 0.23 (0.4), and jawline 0.09 (0.2) (Table 1, Figure 4). The increase in fixation count during the 8 s image exposure interval reflects the increased time observers had available to inspect the seven facial regions.

**TABLE 1:** Overall analysis irrespective of age and gender of the visual stimulus type and the respective rank across all facial regions investigated by total fixation count for the 1‐ and 8‐s image exposure interval. Bold values indicate the mean value, whereas standard deviation is indicated in brackets (SD).

Area	1 s: Overall		8 s: Overall	
Frontal	5.	**0.11** (0.2)	4.	**1.70** (2.2)
Periorbital	1.	**2.33** (1.0)	1.	**12.24** (3.8)
Nose	2.	**0.59** (0.6)	3.	**3.01** (2.8)
Cheeks	4.	**0.19** (0.3)	5.	**0.98** (1.1)
Perioral	3.	**0.35** (0.8)	2.	**3.10** (2.8)
Chin	6.	**0.03** (0.1)	6.	**0.23** (0.4)
Jawlines	7.	**0.01** (0.1)	7.	**0.09** (0.2)

### 3.3 Sex‐Specific Analyses

**FIGURE 5:**
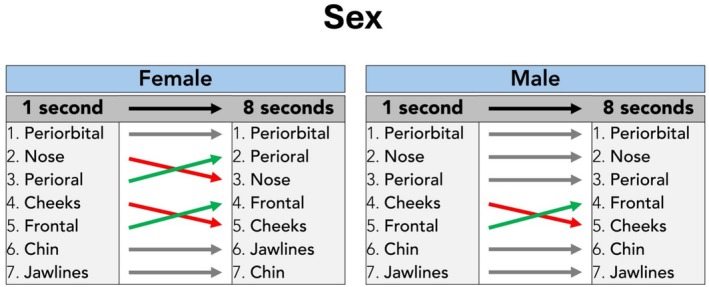
Sex‐dependent comparison of total fixation counts per facial region in 1‐s versus 8‐s image exposure intervals.

When comparing the fixation count between the male and the female visual stimulus images (independent of their age; young and mature grouped together), at 1 s image exposure time, no sex‐specific differences were identified, resulting in the following ranking of facial regions (ranked by highest to lowest fixation count): periorbital, nose, perioral, cheeks, frontal, chin, and jawlines. When presenting the same (but randomized) images for 8 s, it was identified that the periorbital region received the highest fixation in both sexes. For males, the ranking identified at 8 s remained unchanged when compared to the 1 s image exposure time, except for the fourth rank, which changed from cheeks to frontal (frontal one rank up). For females, however, the ranking changed for perioral (one rank up), frontal (one rank up), and jawlines (one rank up) when compared to the 1 s image exposure time (Table 2, Figure 5).

**TABLE 2:** Gender‐specific analysis of the visual stimulus images and the respective rank across all facial regions investigated by total fixation count for the 1‐ and 8‐s image exposure interval. Bold values indicate the mean value, whereas standard deviation is indicated in brackets (SD).

Area	1 s: Female		1 s: Male		8 s: Female		8 s: Male	
Frontal	5.	**0.15** (0.4)	5.	**0.06** (0.2)	4.	**1.53** (3.3)	4.	**1.86** (1.8)
Periorbital	1.	**2.41** (0.9)	1.	**2.26** (1.4)	1.	**12.33** (5.2)	1.	**12.16** (5.2)
Nose	2.	**0.49** (0.6)	2.	**0.68** (0.9)	3.	**2.89** (2.7)	2.	**3.14** (3.4)
Cheeks	4.	**0.21** (0.5)	4.	**0.17** (0.3)	5.	**1.14** (1.3)	5.	**0.82** (1.2)
Perioral	3.	**0.38** (1.1)	3.	**0.32** (0.6)	2.	**3.11** (3.0)	3.	**3.09** (2.9)
Chin	6.	**0.03** (0.1)	6.	**0.03** (0.2)	6.	**0.16** (0.4)	6.	**0.30** (0.5)
Jawlines	7.	**0.02** (0.1)	7.	**0.01** (0.1)	7.	**0.07** (0.2)	7.	**0.11** (0.3)

### 3.4 Age‐Specific Analyses

**FIGURE 6:**
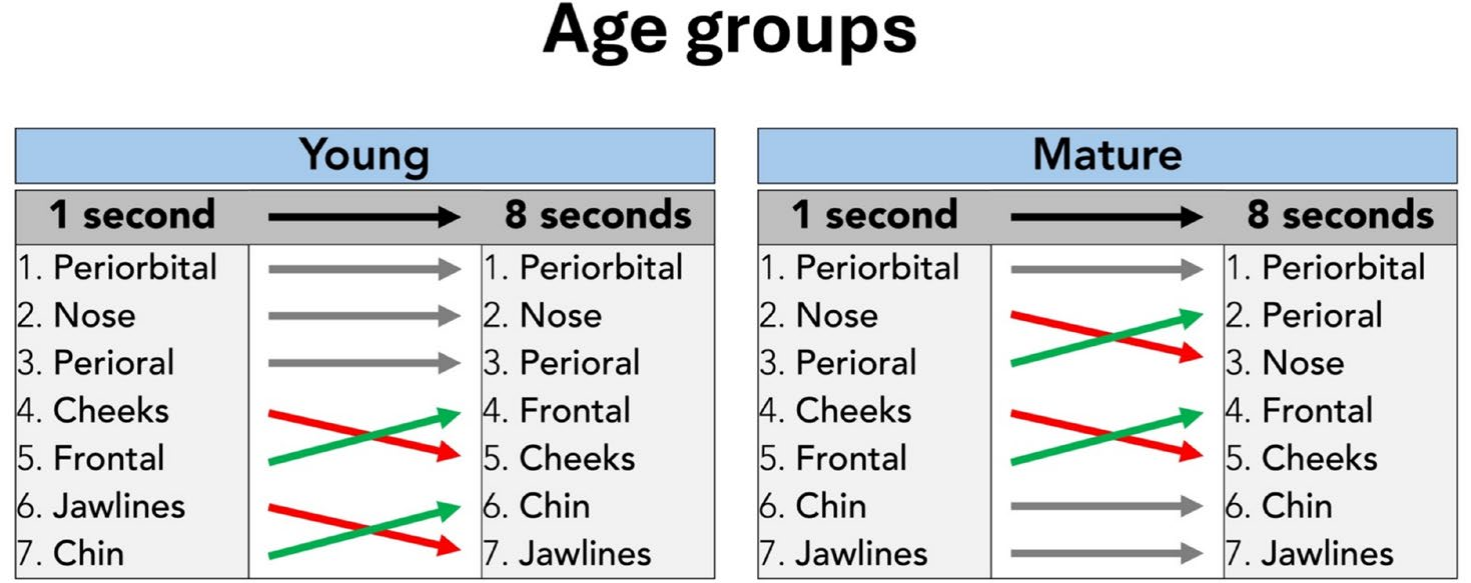
Age‐dependent comparison of total fixation counts per facial region in 1‐s versus 8‐s image exposure intervals.

When comparing the fixation count between young and mature visual stimulus images (independent of their sex; male and female grouped together), at 1 s image exposure time, no age‐specific differences were identified between rank one and five, resulting in the following ranking of facial regions (ranked by highest to lowest fixation count): periorbital, nose, perioral, cheeks, and frontal. When presenting the same (but randomized) images for the duration of 8 s, it was identified that for the young facial images, the ranking remained unchanged for the first three ranks: periorbital, nose, and perioral. However, for mature visual stimulus images, the perioral region changed its rank from rank 3 at 1 s to rank 2 at 8 s (Table 3, Figure 6).

**TABLE 3:** Age‐specific analysis of the visual stimulus images and the respective rank across all facial regions investigated by total fixation count for the 1‐s and 8‐s image exposure interval. Bold values indicate the mean value, whereas the standard deviation is indicated in brackets (SD).

Area	1 s: Young		1 s: Mature		8 s: Young		8 s: Mature	
Frontal	5.	**0.08** (0.2)	5.	**0.13** (0.4)	4.	**1.40** (1.7)	4.	**1.99** (3.0)
Periorbital	1.	**2.27** (1.2)	1.	**2.39** (1.1)	1.	**11.61** (4.1)	1.	**12.87** (4.4)
Nose	2.	**0.55** (0.7)	2.	**0.63** (0.7)	2.	**3.29** (3.0)	3.	**2.74** (3.0)
Cheeks	4.	**0.21** (0.3)	4.	**0.17** (0.5)	5.	**1.24** (1.5)	5.	**0.72** (1.2)
Perioral	3.	**0.35** (1.0)	3.	**0.36** (0.8)	3.	**2.76** (2.6)	2.	**3.45** (3.3)
Chin	7.	**0.01** (0.1)	6.	**0.05** (0.2)	6.	**0.23** (0.4)	6.	**0.23** (0.5)
Jawlines	6.	**0.03** (0.1)	NI	**0.00** (0.0)	7.	**0.10** (0.3)	7.	**0.09** (0.3)

Abbreviation: NI, not inspected.

### 3.5 Age‐ and Sex‐Specific Analyses Combined

**FIGURE 7:**
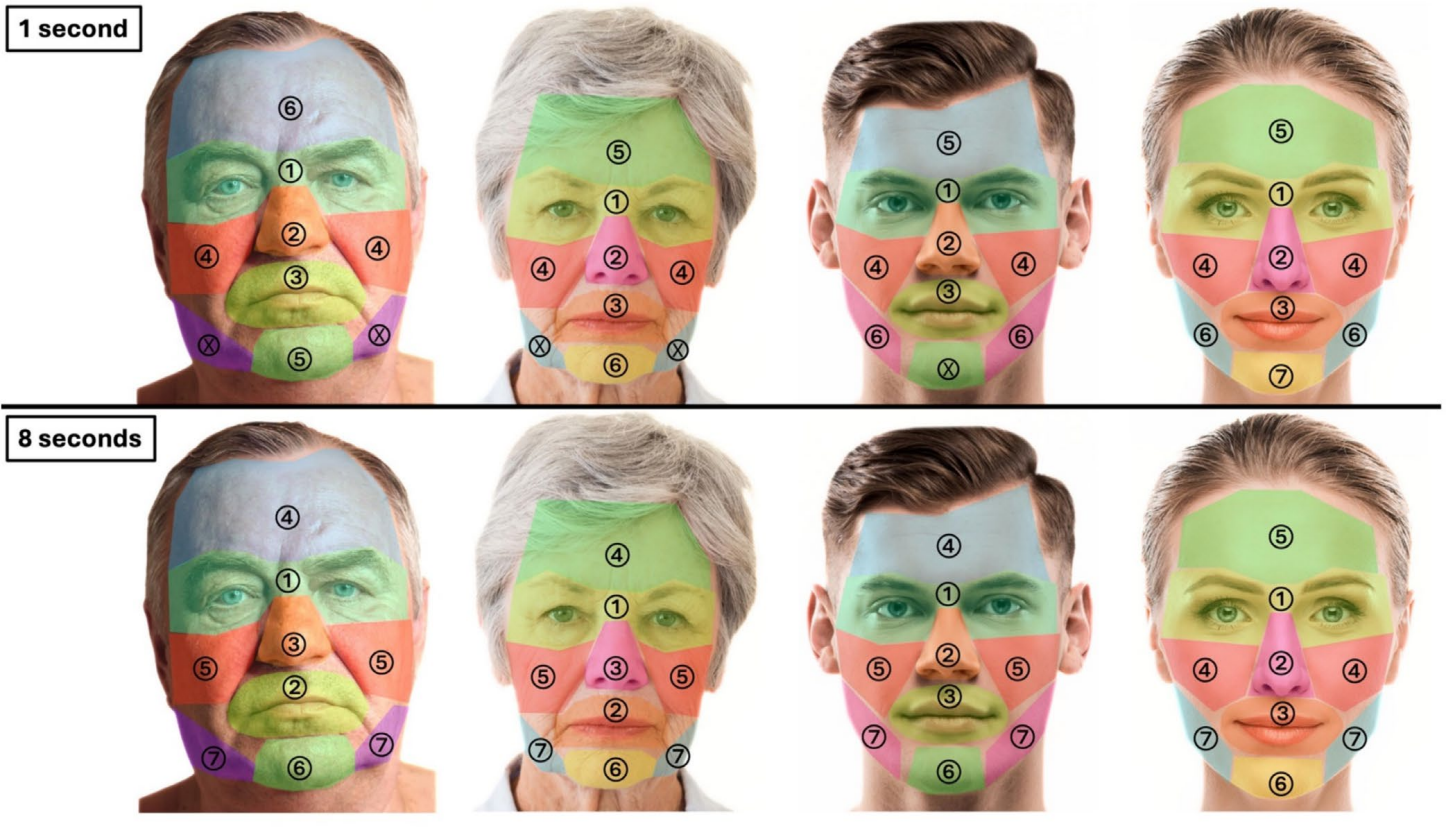
Summary of the total fixation count rankings for all four visual stimulus images in 1‐s and 8‐s image exposure intervals.

**FIGURE 8:**
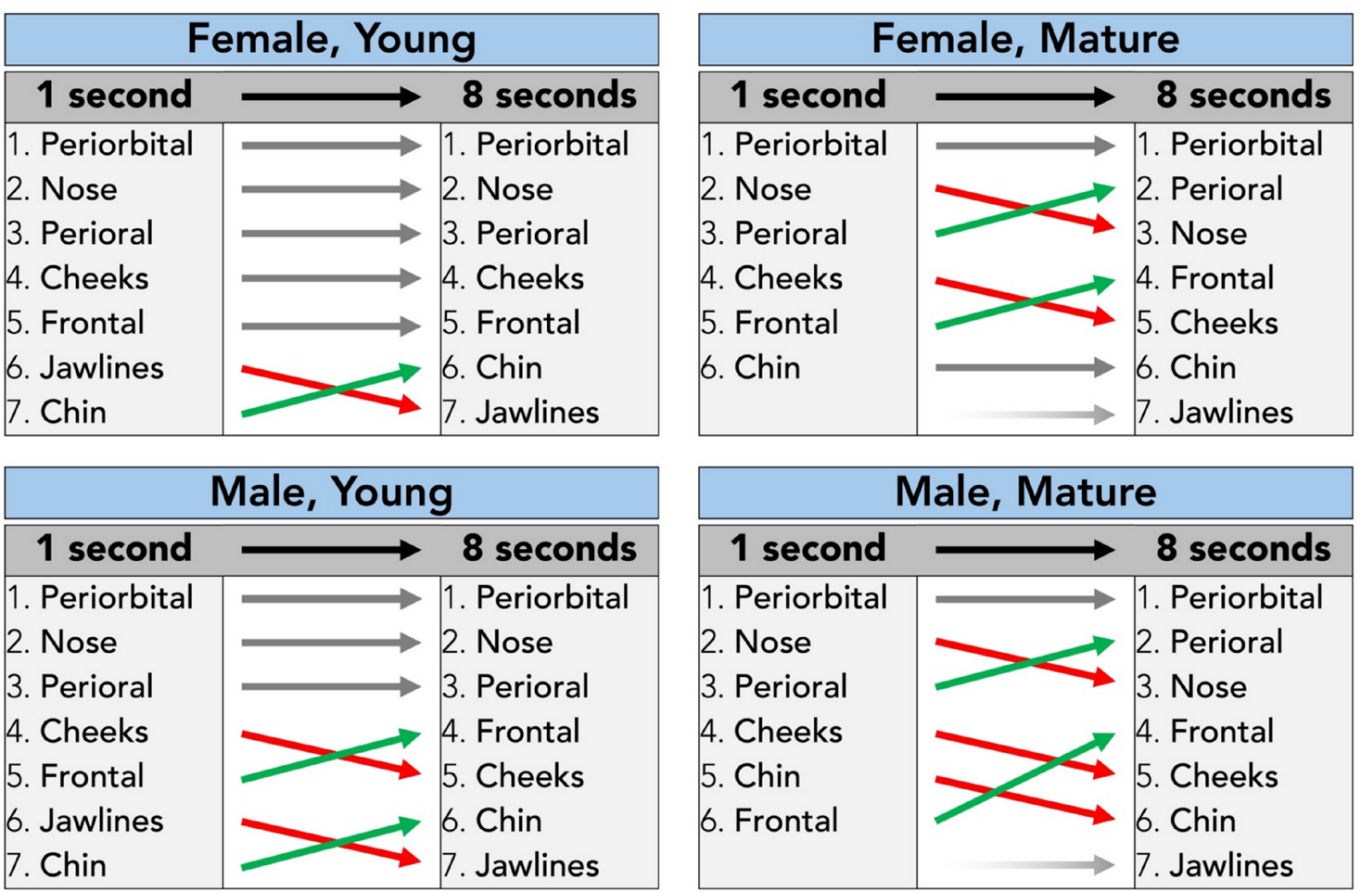
Comparison of total fixation counts per facial region in 1‐s versus 8‐s image exposure intervals, stratified by both age and sex.

When analyzing the fixation count stratified by age and sex combined, it was noted that the highest fixation count per facial region was throughout all categories the periorbital region. In mature facial images, the second most frequently inspected facial region shifted from nose (rank two at 1 s) to perioral (rank two at 8 s). In general, it was identified that the fourth most frequently inspected facial region was during the 1 s interval the cheeks, whereas at 8 s it was the frontal region. Jawline ranked most frequently seventh and hence last position during the 8 s visual stimulus exposure interval, whereas it was not inspected by any of the observers for mature visual stimulus during the 1 s exposure interval (Table 4, Figures 7 and 8).

**TABLE 4:** Age and gender‐specific analysis of the visual stimulus images and the respective rank across all facial regions investigated by total fixation count for the 1‐ and 8‐s image exposure interval. Bold values indicate the mean value, whereas standard deviation is indicated in brackets (SD).

Area		1 s: Young female		1 s: Mature female		1 s: Young male		1 s: Mature male		8 s: Young female		8 s: Mature female		8 s: Young male		8 s: Mature male
Frontal	5.	**0.09** (0.3)	5.	**0.22** (0.7)	5.	**0.07** (0.3)	6.	**0.04** (0.3)	5.	**1.45** (2.8)	4.	**1.60** (4.2)	4.	**1.35** (1.6)	4.	**2.38** (3.0)
Periorbital	1.	**2.51** (1.1)	1.	**2.31** (1.2)	1.	**2.04** (1.6)	1.	**2.47** (1.5)	1.	**11.75** (5.0)	1.	**12.91** (7.3)	1.	**11.47** (6.5)	1.	**12.84** (5.2)
Nose	2.	**0.47** (0.7)	2.	**0.51** (0.8)	2.	**0.62** (1.0)	2.	**0.75** (1.0)	2.	**3.49** (3.8)	3.	**2.29** (2.4)	2.	**3.09** (3.2)	3.	**3.18** (4.1)
Cheeks	4.	**0.20** (0.5)	4.	**0.22** (0.8)	4.	**0.22** (0.5)	4.	**0.13** (0.3)	4.	**1.55** (1.9)	5.	**0.73** (1.2)	5.	**0.93** (1.8)	5.	**0.71** (1.5)
Perioral	3.	**0.42** (1.4)	3.	**0.35** (1.0)	3.	**0.27** (0.7)	3.	**0.36** (0.7)	3.	**2.84** (3.0)	2.	**3.38** (3.4)	3.	**2.67** (3.1)	2.	**3.51** (3.6)
Chin	7.	**0.02** (0.1)	6.	**0.04** (0.2)	NI	**0.00** (0.0)	5.	**0.05** (0.3)	6.	**0.15** (0.6)	6.	**0.16** (0.5)	6.	**0.31** (0.6)	6.	**0.29** (0.7)
Jawlines	6.	**0.04** (0.2)	NI	**0.00** (0.0)	6.	**0.02** (0.1)	NI	**0.00** (0.0)	7.	**0.09** (0.3)	7.	**0.05** (0.2)	7.	**0.09** (0.4)	7.	**0.13** (0.5)

Abbreviation: NI, not inspected.

## 4 Discussion

The aim of this eye tracking‐based study was to investigate differences in perception when the visual stimulus exposure time is varied between 1 and 8 s. The total fixation count per facial region was used as a surrogate parameter to identify facial regions of greater or lesser relevance for facial perception. This investigative approach is different when compared to previous eye tracking studies in which the exposure time was predetermined to 8 s and the duration of stable eye fixations was evaluated. However, observers (including patients) often evaluate faces in much shorter time, e.g., when they scroll through the treatment provider's galleries or when they are presented with images showing such results during consultations. It seems to be important to know if gaze patterns during such activity are different and how they address sex and age‐related features, which are often targeted by aesthetic providers. Given that in the present study the image exposure time was the independent variable and included 1 s versus 8 s, no additional time variable could be used as an outcome parameter as was done in previous studies [12, 13, 14, 15, 16]. Therefore, a time‐independent parameter had to be used in this study to identify gaze patterns and preferences, resulting in the employment of the frequency count of stable eye fixations lasting longer than 0.08 s per pre‐defined facial regions.

The results revealed that even during an inspection time of just 1 s, a stable pattern of facial scanning was identifiable which followed an interesting sequence: periorbital, nose, perioral, cheeks, frontal, chin, and jawline. This pattern is in line with previous studies which have identified that the central facial oval is scanned first: starting with the eyes, then followed by midline structures (nose, perioral), then increasing the radius of the facial scanning to more lateral (cheeks) and more vertical facial regions (frontal, chin), and ending with the most peripheral facial regions that could be observed in frontal facial images (jawline) [18, 19, 20, 21, 22, 23]. The above‐described pattern did not differ majorly when the inspection time was increased to 8 s image exposure time, which could be an indicator that facial scanning follows a certain pattern. However, it needs to be noted that a general shift towards facial regions more important for facial expressions and showing signs of aging (i.e., perioral and frontal region) was observed. This might suggest that during the first impression, rather central facial features (i.e., periorbital, nose, perioral) are observed while the gaze is expanded, and the focus is more directed towards mimically important facial regions (Figure 4).

When looking at the results obtained from the comparison between young versus mature visual stimulus images, no differences were detected between 1 and 8‐s image exposure time for the ranks 1 to 3: periorbital, nose, and perioral (Figure 6). This showcases that the central facial “T” is inspected first and maintains its relevance even if longer viewing times are allowed. However, when mature facial images are presented, the perioral region increases in ranking (from rank 3 to 2) which was detected in both male and female observers when stratification analyses were conducted. This finding is in line with a previous publication by Frank et al. [13] which identified that the perioral region is of great importance in mature aesthetic patients. The authors explained their eye tracking findings with the internal blueprint of beauty theory which is triggered by the plethora of perioral age‐related changes including upper lip lengthening, perioral lines, jowls, pre‐jowl sulcus deformities, labiomandibular sulcus formation, labial thinning, and labial inversion [24, 25, 26, 27, 28]. A similar effect could be observed in the present study in which mature facial images, but not young facial images, resulted in an increase in fixation count between the 1 and the 8 s image exposure time, prompting a change in ranking.

It is to note that the periorbital region was always ranked number 1 independent of image exposure time or visual stimulus (male vs. female, young vs. mature) presented. The importance of looking at the eyes was explained earlier by Kobayashi et al. when comparing the human eye to the eyes of other primates which lack the white scleral triangles surrounding the iris. This contrast between white and dark (or darker) allows for fast non‐verbal communication in proximity to predators or other enemies; this was considered an evolutionary advantage which seems to be imprinted in human behavior [29].

Looking at the remainder of the results of this study, it can be detected that facial regions with greater age‐related changes increase in fixation count at 8 s: perioral, frontal, and jawline. These facial regions are known to change the facial appearance with the formation of rhytids and depressions (forehead) or with facial sagging and an increase in sulcus severity (jawline and labiomandibular sulcus). Opposed to these facial regions, the remainder of the investigated facial regions are known to display fewer age‐related changes like the nose, cheeks, or the chin. This is in line with the internal blueprint of beauty theory, which attracts more attention to facial regions that do not match an individual beauty standard and therefore require more visual information to understand what is inspected. Clinically, this means that facial regions affected by age‐related changes attract the observer's attention more than other facial regions and become therefore (almost) the center of attention. It has been reported that aesthetic surgery procedures alter patterns of observers' attention when viewing a face, decreasing the time spent looking at the typical signs of aging [30]. In this study, we sought to explore whether this attention shift is influenced by the duration of image exposure. This insight is crucial when consulting patients about aesthetic procedures. Our findings suggest that when presenting patients with images depicting the effects of a procedure on aging individuals—particularly in the frontal region, chin, or jawline—extending the image exposure time ensures that observers thoroughly focus on these critical areas.

However, this study is not free of limitations. For instance, a larger sample size would have allowed for more robust conclusions. Dedicated studies focusing on comparisons between medical and non‐medical observers, with specific distinction for those who are involved in plastic surgery or aesthetic medicine, would be valuable in providing deeper insight to broaden the scope on how professional background influences facial perception. Furthermore, all assessments were made by observers of Polish origin with a Caucasian ethnic background. This homogeneity might introduce potential biases, as cultural influence plays a significant role in facial perception and the assessment of attractiveness, thereby limiting the applicability of our findings to other ethnic groups and cultural backgrounds. Future research should aim to include observers from various ethnic and cultural backgrounds to substantiate and expand upon the findings presented. Further, it needs to be validated whether the findings obtained herein also apply to observers with experiences in the fields of plastic surgery or aesthetic medicine.

## 5 Conclusion

The results indicated that even with an inspection time of just 1 s, a stable facial scanning pattern emerged: starting with the central facial oval (i.e., periorbital, nose, perioral region), and gradually expanding laterally (cheeks) and vertically (forehead, chin, and jawline). This pattern remained consistent with an 8‐s inspection time, suggesting a structured facial scanning process. However, a shift towards regions crucial for facial expressions and age‐related changes (perioral and forehead) was noted upon longer image exposure intervals, implying that initial impressions focus on central features while extended observation emphasizes mimically important areas. Finding that observers (potential patients) focus on different facial aspects depending on observation duration should be of significant interest to aesthetic medicine experts and plastic surgeons. These results, obtained through eye‐tracking analyses, underscore the importance of patient‐directed aesthetic assessments. They also emphasize the need for enhanced communication with patients by presenting images that illustrate the potential effects of procedures on various facial regions.

## Author Contributions

M.A., S.C., J.K., A.K.T., B.A., and L.K. conceptualized the study and contributed to the study design. M.A., S.K., J.K., and A.K.T. performed the data collection and conducted the experiments. S.C., R.J.R., J.K., S.M.S. and C.B. assisted in refining the experimental protocol and provided critical technical or clinical insights. M.A., S.C., S.K., D.W. and B.A. supported statistical analysis and data validation. M.A., S.M.S., S.C., L.K., K.D. and J.B.G. interpreted the results and contributed to drafting the manuscript. R.J.R., S.M.S., L.K., C.B., B.A. and K.D. provided major revisions and critical feedback on the intellectual content. All authors contributed to manuscript and approved the final version of the manuscript.

## Ethics Statement

Ethical approval was obtained from the Bioethics Committee of the Medical University of Łódź, protocol number RNN/217/22/KE.

## Conflicts of Interest

The authors declare no conflicts of interest.

## Data Availability

The data that support the findings of this study are available from the corresponding author upon reasonable request.
